# Primary cardiac lymphoma in HIV infected patients: A clinicopathological report of two cases

**DOI:** 10.1016/j.amsu.2021.102757

**Published:** 2021-08-23

**Authors:** Moshawa Calvin Khaba, Makenga Fidele Kampetu, Mamokgethi Christina Rangaka, Mohamed Karodia, Shere Peter Ramoroko, Elelwani Innocentia Madzhia

**Affiliations:** a Department of Anatomical Pathology, Dr George Mukhari Academic Laboratory, National Health Laboratory Service, Sefako Makgatho Health Sciences University, South Africa; b Department of Cardiothoracic Surgery, Dr George Mukhari Academic Hospital, Sefako Makgatho Health Sciences University, South Africa; c Department of Paediatric Cardiology, Dr George Mukhari Academic Hospital, Sefako Makgatho Health Sciences University, South Africa; d Department of Clinical Hematology, Dr George Mukhari Academic Hospital, Sefako Makgatho Health Sciences University, South Africa; e Department of Paediatric Oncology, Dr George Mukhari Academic Hospital, Sefako Makgatho Health Sciences University, South Africa

**Keywords:** Primary cardiac lymphoma, Diffuse large b-cell lymphoma, Burkitt lymphoma, HIV/AIDS

## Abstract

**Introduction and importance:**

Due to advances in diagnostic methods and human immunodeficiency virus, there has been a recent increase in cardiac involvement by lymphoma.

**Case presentation: case 1:**

15-year-old HIV infected male patient presented with features of heart failure and cardiac tamponade. The transthoracic echocardiogram showed pericardial effusion and a right atrioventricular mass. The resected tumour was confirmed to be diffuse large b-cell lymphoma on histopathology. Unfortunately, the patient died few hours after surgery. Case 2: 30-year-old HIV infected pregnant female presented with features of cardiac tamponade. The transthoracic echocardiogram showed pericardial effusion with right atrial mass. The resected tumour was confirmed to be Burkitt's lymphoma on histopathology. She was successfully treated with chemotherapy.

**Clinical discussion:**

Cardiac lymphomas are rare with most cases diagnosed on autopsy. However, advances in diagnostic methods has increased antemortem diagnosis with subsequent optimal management. Majority of the cases are of B-cell lineage, although T-cell origin has been reported.

**Conclusion:**

A high index of suspicion of cardiac lymphoma should be maintained in the right clinical setting in order to receive adequate attention and management.

## Highlights

• Primary cardiac lymphomas (PCL) are rare and aggressive tumours.
• PCL from an African perspective has not been described.
• Early diagnosis maybe associated with good clinical outcome.
• High index of suspicion in the right clinical setting should be maintained.

## 1 Introduction

Cardiac masses are composed of tumours, thrombi and vegetations [1]. Majority of the tumours are benign which comprises approximately 90% of primary cardiac tumours while 10% are malignant [1,2]. The common malignant tumours are lymphomas and sarcomas [2]. Cardiac lymphomas are rare with associated poor clinical outcomes [2,3]. Therefore, it is imperative that they are diagnosed early to allow accurate management [[2], [3], [4]]. Herein, we present two cases diagnosed with cardiac lymphoma who presented with signs and symptoms of obstruction. These case reports seek to enhance the knowledge on cardiac lymphoma, which will enable clinicians to diagnose them with less difficulty and possibly develop guidelines on management of these cases.

This manuscript has been reported in line with SCARE 2020 criteria [5].

## 2 Case presentations

We report two cases with primary cardiac lymphomas (Table 1).

**Table 1:** Clinicopathological features.

Features	Patient 1	Patient 2
Age	15	30
Gender	Male	Female
Co-morbidities	HIV positive	HIV positive
Presentation	Cardiac tamponade	Cardiac tamponade
Echo	Pericardial effusion with features of cardiac tamponade and right atrial mass	Pericardial effusion with right atrial mass which prolapsed into the right ventricle through to the tricuspid valve
Diagnosis	Diffuse large B-cell lymphoma	Burkitt lymphoma
FISH study	Negative	MYC translocation
Chemotherapy	n/a	CODOX-M/IVAC
Outcome	Died	Recovered, in remission

## 3 Case 1

15-year-old male patient who presented with dyspnea at rest, night sweats, joint pains, puffy face and swollen legs. He was positive for human immunodeficiency virus (HIV) positive through vertical transmission and currently on antiretroviral therapy (ART) with CD4 T lymphocytes of 430 cell/uL and viral load of 2906 copies/mL. On general examination, he was emaciated with bipedal oedema, distended neck veins and generalized significant lymphadenopathy. He had a respiratory rate of 25 breaths per minute (bpm), heart rate of 110 beats per minute (bpm) and blood pressure (BP) of 80/60 mmHg.

On cardio-respiratory examination, the air entry was decreased on the left lower zones with bilateral crepitations indicative of left pleural effusion. He had a displaced apex beat with muffled heart sounds and no audible murmur. The abdomen was distended with ascites and hepatosplenomegaly. The chest x-ray (CXR) showed a globular heart with patchy infiltrations and left pleural effusion.

Transthoracic echocardiogram (TTE) showed a massive pericardial effusion with features of cardiac tamponade and right atrial mass.

The clinical diagnosis was that of right cardiac tumour complicated by cardiac tamponade. He was started on solucortef. Furthermore, an urgent left anterior thoracotomy for pericardial window biopsy with resultant cardiac tamponade relief was performed. However, he remained unstable and the repeat TTE showed right ventricular inflow obstruction. He underwent urgent tumour resection with the cardiopulmonary bypass assistance. He unfortunately died within 24 hours post-operatively.

Multiple friable fragments of tissue with a combined measurement of 70 x 80 × 25mm were received for histopathological assessment. Microscopic examination showed tumour tissue exclusively which was arranged in sheets. The tumour cells were large with eosinophilic cytoplasm, pleomorphic and vesicular nuclei with 1–2 prominent nucleoli. Brisk Mitotic activity and apoptosis were noted. The tumour cells were positive for CD45, CD20 and CD10 while EBV, CD3, MUM-1, bcl-2 and bcl-6 were negative (Fig. 3A–D). Fluorescence in situ hybridization (FISH) was negative for rearrangement of the MYC gene. The overall features were in keeping with diffuse large b-cell lymphoma.

## 4 Case 2

30-year-old pregnant female patient at 24 weeks of gestation was referred from obstetrics with a one-week history of progressive dyspnea, non-productive cough and swelling of legs. She was HIV positive on ART with CD4 T lymphocytes of 356 cell/uL and low detectable viral load.

On general examination, she was in respiratory distress with a BP of 88/59 mmHg and tachycardia of 137 bpm. Cardio-respiratory revealed distended neck veins, pericardial rub, muffled heart sounds without audible murmurs. She also had bilateral crepitations. A gravid uterus was confirmed.

**Fig. 1:**
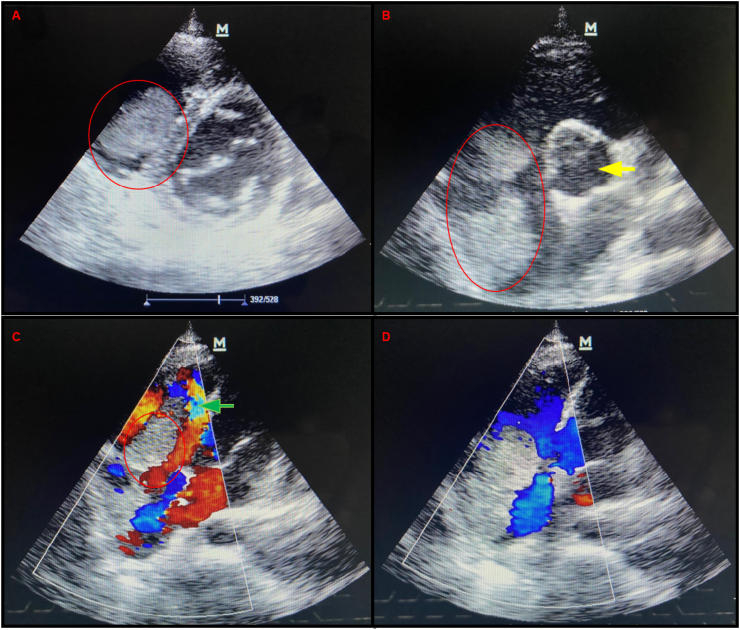
Echocardiogram. A – D shows right atrial tumour protruding through the tricuspid valve (TV) into the RV causing TV regurgitation. Red circles show tumour in the right atrium attached to the TV. Yellow arrow shows aorta in short axis. The green arrow shows mosaicism pattern due to TV incompetence. (For interpretation of the references to colour in this figure legend, the reader is referred to the Web version of this article.)

The CXR showed cardiomegaly with globular heart and diffuse bilateral lung infiltrates. TTE revealed a large pericardial effusion with right atrial mass which prolapsed into the right ventricle through to the tricuspid valve (Fig. 1A–D).

The differential clinical diagnosis for the cardiac mass included atrial myxoma, thrombus and vegetations complicated by cardiac tamponade.

An urgent pericardiocentesis was performed with resultant clinical improvement. Once the patient was optimized, an urgent tumour resection was perfomed with cardiopulmonary bypass.

The surgery was uneventful and the patient was transferred to intensive care unit (ICU) where she was extubated the same day. Furthermore, she had spontaneous abortion on day 2 post-surgery.

**Fig. 2:**
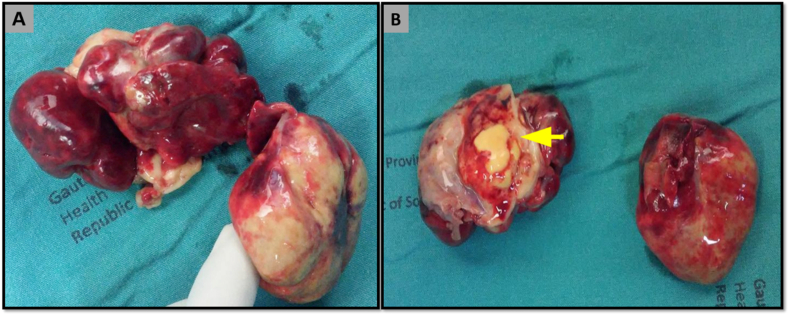
A–B: Gross features show hemorrhagic and multinodular tumour with central necrosis and pus (yellow arrow). (For interpretation of the references to colour in this figure legend, the reader is referred to the Web version of this article.)

Two pieces of tumours were received for histopathological assessment. The larger tumour measured 60 x 35 × 25mm and smaller 40 x 40 × 17mm. On cut section, they were fleshy with necrosis and pus (Fig. 2A and B).

**Fig. 3:**
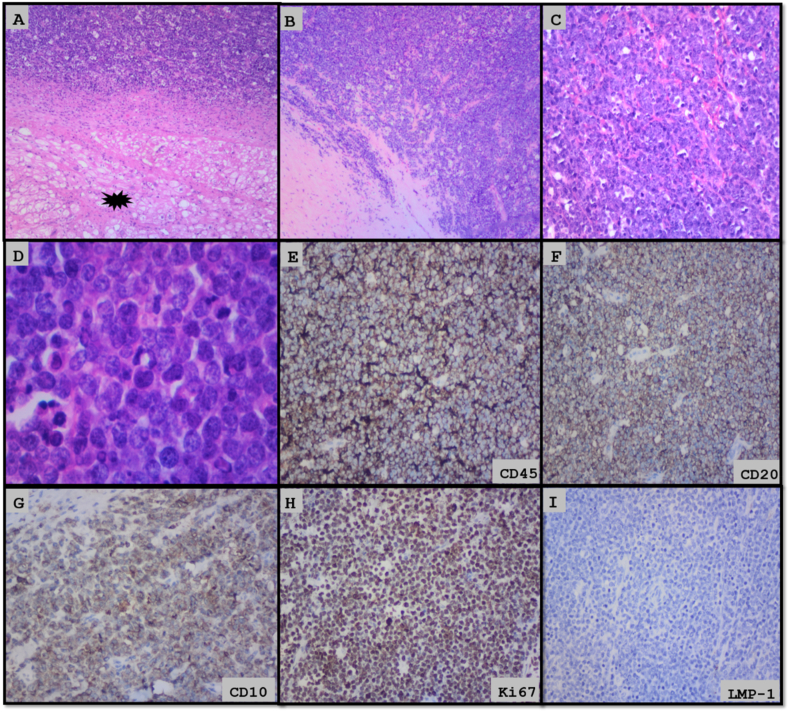
A–D: Show infiltrating lymphoid tumour with starry sky appearance. Black star arrow shows myocardial invasion; E − I (immunohistochemistry) show positive CD45, CD20 and CD10, high proliferation index (Ki67) and negative EBV (LMP-1).

Microscopically, both specimen show similar features derived from the cardiac muscle with an invasive tumour with diffuse pattern and extensive coagulative tumour necrosis. A “starry sky” appearance was seen. The tumour cells were intermediate and large with amphophilic cytoplasm, pleomorphic and vesicular nuclei with multiple peripherally located nucleoli and delicate nuclear membrane. There was brisk mitotic activity with Ki67 proliferation index of approximately 98%. The tumour cells were positive for CD45, CD20 and CD10 while EBV, CD3, MUM-1, bcl-2 and bcl-6 were negative (Fig. 3A–I).

**Fig. 4:**
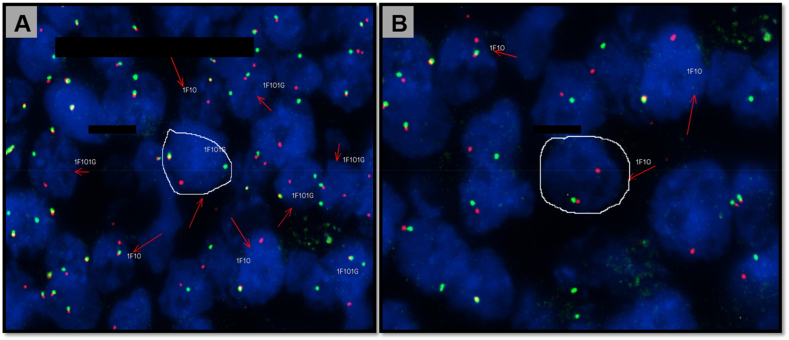
A–B: FISH study shows MYC translocation by break-apart probe (white circles with separate green and red signals). (For interpretation of the references to colour in this figure legend, the reader is referred to the Web version of this article.)

FISH studies confirmed translocation t(8; 14)(q24; q32) (Fig. 4A and B) while bcl2 and bcl6 translocation were not evident. These features were consistent with Burkitt's lymphoma.

Upon receipt of histopathological diagnosis, the patient received 4 cycles of cyclophosphamide, vincristine, doxorubicin, high-dose methotrexate/ifosfamide, etoposide, high-dose cytarabine (CODOX-M/IVAC). She tolerated and responded well to this regimen. On follow-up visit she was well and in remission. She's currently being followed up by cardiothoracic and hematology departments.

## 5 Discussion

The association of Epstein-Barr virus–related lymphoproliferative disorders and HIV/AIDS has contributed to the increase in the prevalence of cardiac lymphomas [6,7]. However, cardiac involvement by lymphoma is rare and to date it has been documented in 10–25% of autopsy cases with the largest autopsy series performed by Chinen K and colleagues which consisted of only 25 cases [8].

Despite this, progress in diagnostic methods in recent times has contributed in discovery of more antemortem cases with increase in knowledge of this entity and better management [8,9].

Cardiac lymphoma (CL) comprises approximately 1–2% of all cardiac tumour [3,10] and 0.5% of extra-nodal lymphomas [3,4]. They are aggressive and fatal tumours which usually manifest after the fifth decade of life and show slight male predominance [4,10].

CL is divided in primary and secondary which is made possible by complete clinical and radiological assessment. Primary lymphoma exclusively involves the heart or pericardium. Furthermore, other diagnostic criteria include primary burden of tumour is within the pericardium or heart despite the presence of extra-cardiac disease; and the clinical picture is directly proportional to the impact of lymphoma on heart structure or function [7,11]. Both our cases met this criteria, and were therefore regarded as primary cardiac lymphoma (PCL).

The clinical presentation is heterogenous [12] and depends on the site of involvement of the heart, size and growth rate [3]. The right atrium is the most affected chamber [10,12,13]. The common features include heart failure, arrhythmia, valvular disease, myocardial infarct, tamponade and outflow obstruction [4,[7], [8], [9], [10]]. Superior vena cava syndrome has also been described [4].

Chest x-ray, *trans*-thoracic or *trans*-esophageal echocardiography (TTE/TEE), computed tomography (CT) and magnetic resonance imaging (MRI) are helpful in establishing the diagnosis. Moreover, they should increase the high index of suspicious cases. CXR usually reveals cardiomegaly and/or pleural effusion.

Whilst TEE provides satisfactory images of the right atrium; TTE has a higher sensitivity for primary cardiac tumours [9,13].

CT and MRI can easily visualize the cardiac tumours and great vessels [13], however, the urgent nature of these cases may not allow for these studies to be undertaken. This was evident in both our cases as they needed emergency pericardiocentesis and subsequent surgery for symptoms relief and diagnostic purpose.

Definite diagnosis is made on histopathological assessment of the mass. However, cytological examination of pericardial fluid can be used in the diagnosis [10].

Grossly, cardiac lymphomas are firm and white single or multiple tumours which are infiltrative and intramural. Furthermore, they can also involve the epicardium and pericardium. Microscopically, cardiac lymphomas are usually of B-cell type with DLBCL as the most common type followed by follicular lymphoma, plasmablastic lymphoma, small lymphocytic lymphoma and Burkitt lymphoma [7,11](4,9). T-cell lymphomas have also been reported [4,10].

The standardized therapy for cardiac lymphoma has not yet being established whilst the role of surgery is limited [3,10]. Surgical treatment may help by temporarily relieving the obstruction to provide time for chemotherapy to achieve its therapeutic effect(3).

PCL responds well to chemotherapy and the regimen depends on the type of lymphoma. DLBCL responds well to cyclophosphamide, doxorubicin, vincristine and prednisone (CHOP) [3]. Adding rituximab to this regimen has shown to increase the overall survival rate [9,11]. Burkitt lymphoma responds well to 4 cycles of CODOX-M/IVAC which is what patient 2 received.

Chemotherapy followed by radiotherapy may enhance survival, although its efficacy remains to be determined. The overall response rate of patients with PCL to chemotherapy is 79% and the complete remission rate is 59% [4,9]. CL have poor prognosis with median overall survival of 7–12 months [3,4,9]. Poor prognosis is associated with immunocompromised status, extra-cardiac disease and left ventricular involvement.

## 6 Conclusion

High grade B cell lymphoma of the heart should receive a high index of suspicion in HIV positive patients presenting with a cardiac mass. This will enhance prompt diagnosis with improved clinical outcome.

## Provenance and peer review

Not commissioned, externally peer-reviewed.

## Ethical approval

Sefako Makgatho University Research Ethics Committee(SMUREC) approved the publication of this case report. SMUREC/M/96/2021.

## Sources of funding

None.

## Consent

Written informed consent was obtained from the patient for publication of this case report and accompanying images. A copy of the written consent is available for review by the Editor-in-Chief of this journal on request.

## Author contribution

All authors wrote the case report. Dr MC Khaba organized the manuscript and critically revised the paper.

## Research registration

Not applicable

## Guarantor

Dr MC Khaba.

## Declaration of competing interest

None.
