# Supplementary material for: Primary cardiac lymphoma in HIV infected patients: A clinicopathological report of two cases
Source: Ann Med Surg (Lond). 2021 Aug 23;69:102757. doi: 10.1016/j.amsu.2021.102757 (PMC8387905; doi:10.1016/j.amsu.2021.102757)
Supplement: Multimedia component 1 [file mmc1.docx]

| **SCARE Checklist** | | | |
| --- | --- | --- | --- |
| **Topic** | **Item** | **Checklist item description** | **Page Number** |
| **Title** | **1** | Primary cardiac lymphoma in HIV infected patients: A clinicopathological report of two cases |  |
| **Key Words** | **2** | Cardiac lymphoma, diffuse large b-cell lymphoma, Burkitt lymphoma, cardiac tamponade, HIV/AIDS | 1 |
| **Abstract** | **3a** | Introduction  Due to advances in diagnostic methods and human immunodeficiency virus, there has been a recent increase in cardiac involvement by lymphoma. | 1 |
|  | **3b** | The patient's main concerns and important clinical findings.  Cases presentation:  Case 1 was a 15-year-old HIV infected male patient presented with features of heart failure and cardiac tamponade.  Case 2 was a 30-year-old HIV infected pregnant female presented with features of cardiac tamponade. |  |
|  | **3c** | Case 1: The transthoracic echocardiogram showed pericardial effusion and a right atrioventricular mass. The resected tumour was confirmed to be diffuse large b-cell lymphoma on histopathology. Unfortunately, the patient died few hours after surgery in intensive care unit.  Case 2: The transthoracic echocardiogram showed pericardial effusion with right atrial mass. The resected tumour was confirmed to be Burkitt’s lymphoma on histopathology. She was successfully treated with chemotherapy. |  |
|  | **3d** | Conclusion  A high index of suspicion of cardiac lymphoma should be maintained in the right clinical setting in order to receive adequate attention and management. Prompt diagnosis and management is associated with good clinical outcome. |  |
| **Introduction** | **4** | 90% of primary cardiac tumours are benign while 10% are malignant. Lymphomas and sarcomas are the common malignant tumours. Cardiac lymphomas are rare and associated with poor clinical outcomes. | 2 |
| **Patient Information** | **5a** | Two African patients aged 15 and 30 years old. 1 male and 1 female | 2 - 4 |
|  | **5b** | Both cases presented with cardiac tamponade |  |
|  | **5c** | Both patients were HIV infected |  |
|  | **5d** | **Both patients were on antiretroviral therapy. There was no reported family history of note from both the patients.** |  |
| **Clinical Findings** | **6** | Patients 1  Emaciated with respiratory distress, tachycardia and hypotension.  Bilateral decreased air entry, displaced apex beat with muffled heart sounds  Patient 2:  Respiratory distress, tachycardia and hypotension. Distended neck veins, pericardial rub and muffled heart sounds | 2 - 4 |
| **Timeline** | **7** | Patient 1 died after surgery.  Patient 2 improved dramatically and completed chemotherapy. Currently in remission |  |
| **Diagnostic Assessment** | **8a** | Both patients were done transthoracic echocardiogram which revealed pericardial effusion and cardiac masses. Chest x-ray showed cardiomegaly on both cases. | 2 - 4 |
|  | **8b** | Correct diagnosis were done on histopathology |  |
|  | **8c** | Cardiac myxoma and vegetation were the primary clinical consideration |  |
|  | **8d** | Patient 1 died while Patient 2 responded well to chemotherapy |  |
| **Therapeutic Intervention** | **9a** | Both patients were on oxygen therapy. They underwent pericardiocenthesis and surgery | 2 - 4 |
|  | **9b** | Intravenous fluids and steroids |  |
|  | **9c** | Patient 1 and 2 were initially treated in the medical ward and obstetrics, respectively |  |
|  | **9d** | **The procedure was performed by Dr Ramoroko (senior specialist) assisted by Dr Kampetu (resident).** |  |
|  | **9e** | None |  |
|  | **9f** | **Patient 1 died in intensive care unit.**  **Patient 2 completed chemotherapy and she’s currently being managed as outpatient.** |  |
| **Follow-up and**  **Outcomes** | **10a** | Patient 2: On review after discharge from the hospital, outcomes were satisfactory. | 2 – 4 |
|  | **10b** | Patient 2: Physical examination did not reveal any form of recurrence. She had started gaining some weight. |  |
|  | **10c** | **Patient 2: The patient was adherent to chemotherapy which she tolerated well.**  **Patients 1 died before treatment could be commenced.** |  |
|  | **10d** | Patient 1 died.  Patient 2 is still alive. Currently being followed up as an outpatient without complication |  |
| **Discussion** | **11a** | Cardiac lymphomas are rare and most cases are diagnosed on post-mortem. Advances in diagnostic method has increased antemortem diagnosis. | 5 - 7 |
|  | **11b** | The association of HIV and high grade B-cell lymphoma has seen a recent increase in the diagnosis of cardiac lymphomas with good clinical outcome if diagnosed early. |  |
|  | **11c** |  |  |
|  | **11d** | Early diagnosis improve clinical outcome |  |
| **Patient Perspective** | **12** | **Patient 1 died after surgery.**  **Patient 2: On subsequent follow up, the patient was well and had recovered very well.** | 4 |
| **Informed Consent** | **13** | Written informed consent was obtained from the patient for publication of this case report and accompanying images. A copy of the written consent is available for review by the Editor-in-Chief of this journal on request.  This study received ethics approval from Sefako Makgatho Health Sciences University.  Ref: **SMUREC/M/96/2021** |  |
| **Additional Information** | **14** | None |  |
